# Inverted stereocontrol of iridoid synthase in snapdragon

**DOI:** 10.1074/jbc.M117.800979

**Published:** 2017-07-12

**Authors:** Hajo Kries, Franziska Kellner, Mohamed Omar Kamileen, Sarah E. O'Connor

**Affiliations:** From the ‡ Department of Biological Chemistry, The John Innes Centre, Norwich NR4 7UH, United Kingdom and; § LeafSystems, Norwich NR4 7UH, United Kingdom

**Keywords:** alcohol dehydrogenase (ADH), natural product, natural product biosynthesis, plant biochemistry, terpenoid

## Abstract

The natural product class of iridoids, found in various species of flowering plants, harbors astonishing chemical complexity. The discovery of iridoid biosynthetic genes in the medicinal plant *Catharanthus roseus* has provided insight into the biosynthetic origins of this class of natural product. However, not all iridoids share the exact five- to six-bicyclic ring scaffold of the *Catharanthus* iridoids. For instance, iridoids in the ornamental flower snapdragon (*Antirrhinum majus*, Plantaginaceae family) are derived from the C7 epimer of this scaffold. Here we have cloned and characterized the iridoid synthase enzyme from *A. majus* (AmISY), the enzyme that is responsible for converting 8-oxogeranial into the bicyclic iridoid scaffold in a two-step reduction–cyclization sequence. Chiral analysis of the reaction products reveals that AmISY reduces C7 to generate the opposite stereoconfiguration in comparison with the *Catharanthus* homologue CrISY. The catalytic activity of AmISY thus explains the biosynthesis of 7-epi-iridoids in *Antirrhinum* and related genera. However, although the stereoselectivity of the reduction step catalyzed by AmISY is clear, in both AmISY and CrISY, the cyclization step produces a diastereomeric mixture. Although the reduction of 8-oxogeranial is clearly enzymatically catalyzed, the cyclization step appears to be subject to less stringent enzyme control.

## Introduction

**Figure 1.**
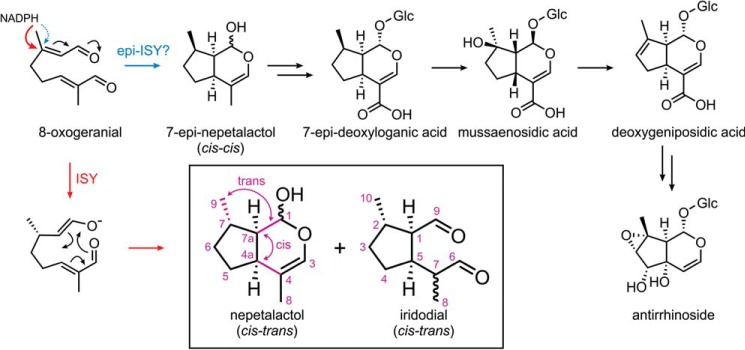
**Proposed epi-ISY step in antirrhinoside biosynthesis.** The biosynthesis of antirrhinoside has been elucidated based on deuterium labeling studies (15–18). Antirrhinoside biosynthesis requires a configuration of the nepetalactol precursor (*blue arrow*) different from that found previously with CrISY (*red arrows*). We hypothesize that an epi-ISY performs the synthesis of 7-epi-nepetalactol in *A. majus*. The CrISY reaction involves hydride transfer from NADPH to generate an enolate intermediate that then cyclizes to various configurational isomers of nepetalactol and iridodial. For the major *cis—trans* nepetalactol and *cis—trans* iridodial product of CrISY, the stereochemical nomenclature and atom numbering are shown in *purple*.

The iridoid glucoside antirrhinoside (Fig. 1) makes up several percent of the dry weight of the common ornamental plant snapdragon (*Antirrhinum majus*) (1–3). In general, iridoids such as antirrhinoside mediate important plant–insect and insect–insect interactions. Plants appear to harness iridoid glucosides to deter herbivores. The herbivore is affected by the toxic dialdehydes liberated by deglycosylation of the iridoid in the injured plant tissue or insect gut (4, 5). Some herbivorous insects can sequester iridoid glucosides and exploit the toxic effect for their own defensive systems (6). Additionally, many iridoid glucosides are believed to have beneficial health properties for humans. Foods such as olives may owe some of their health-promoting properties to iridoid ingredients with anti-inflammatory (7), antimicrobial, and anticancer effects (8).

Because iridoid glucosides are apparently not directed against a specific molecular target, we hypothesize that no specific selection pressure acts to limit the structural diversity of iridoids. On the contrary, in a race of arms with herbivore β-glucosidases evolving away from toxic iridoid glucoside specificity, structural diversity of the iridoid glucoside protoxin may be strongly favored. Accordingly, the usual scope of action of plant secondary metabolism, hydroxylations, acylations, and glycosylations, gives rise to chemotaxonomic variability of the iridoid scaffold down to the species level (9, 10). Additional structural diversity originates from configurational variations of the iridoid core scaffold, which has a fused five- and six-membered ring with multiple stereocenters (Fig. 1).

There has been extensive structural and mechanistic investigation of iridoid synthase (ISY),^3^ the enzyme that creates this core bicyclic scaffold (Fig. 1), from the medicinal plant Madagascar periwinkle (*Catharanthus roseus*). These studies have revealed how the iridoid core cyclizes after transfer of a hydride from NADPH to the linear precursor 8-oxogeranial (11–13). In *Catharanthus*, only iridoids with the stereocenter C7 fixed in the *S* configuration and the ring fusion in the configuration commonly referred to as “*cis–trans*” (Fig. 1; hydrogens *cis* at C4a and C7a and *trans* at C7a and C7) are observed. The hydride transfer step catalyzed by iridoid synthase accounts for the configuration at C7. “Epi-iridoids” with an inverted methyl group at C7 (*e.g.* antirrhinoside, catalpol, epi-loganic acid, penstemoside) are common in Plantaginaceae (10, 14). A detailed biosynthetic hypothesis for 7-epi-iridoids has been developed in Plantaginaceae based on deuterium labeling. Only deuterated 7-epi-deoxyloganic acid, but not deoxyloganic acid, was incorporated into iridoids in *Scrophularia racemosa*, *Plantago major*, and *Buddleja davidii*. Although the C7 stereocenter is removed and reinstalled in later biosynthetic steps, these studies strongly suggest that 7-epi-nepetalactol (C7-*R*) is the productive iridoid intermediate (Fig. 1) (15–18). We hypothesize that an epi-iridoid synthase that reduces C7 of 8-oxogeranial with *R* preference is involved in the biosynthetic pathway. We searched for a homologue of iridoid synthase that performs the *R*-selective reduction of the iridoid precursor 8-oxogeranial. Here we identify the iridoid synthase from *A. majus* (AmISY), which displays epi-iridoid synthase activity.

## Results

### Identification of AmISY

**Figure 2.**
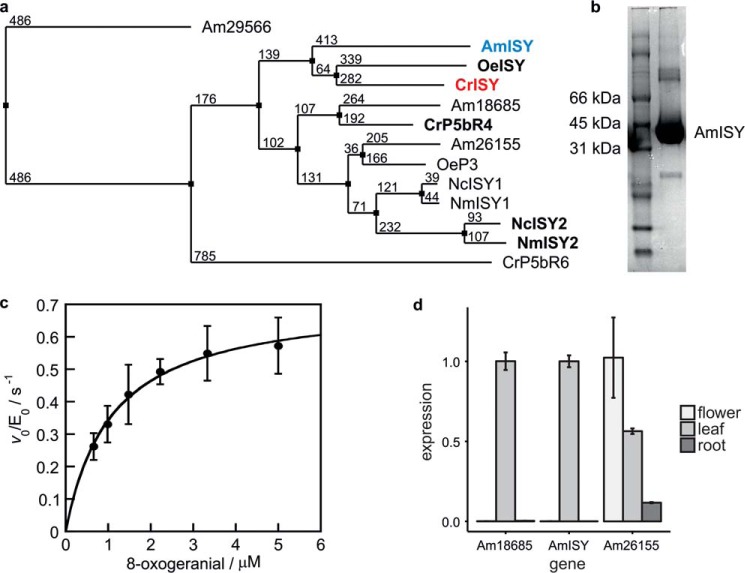
**Identification of AmISY.** *a*, phylogenetic tree of iridoid synthase homologues in *A. majus* (*Am*), *Olea europaea* (*Oe*), *C. roseus* (*Cr*), *Nepeta cataria* (*Nc*), and *N. mussinii* (*Nm*). The neighbor joining tree was built from a MuscleWS alignment using the BLOSUM62 similarity matrix in Jalview 2.10.1 (24). *Numbers* next to the nodes indicate evolutionary distances. Proteins with proven iridoid synthase activity are highlighted in *bold font*. One of the *A. majus* homologues (AmISY or Am18679) groups closely with the iridoid synthases from *O. europaea* and *C. roseus. b*, SDS-PAGE of nickel affinity- and gel filtration chromatography–purified AmISY. *c*, the 8-oxogeranial–dependent NADPH consumption of AmISY showed catalytic parameters close to those of CrISY at a fixed NADPH concentration of 50 μm (AmISY: *k*_cat_ = 0.72 ± 0.02 s^−1^, *K_m_* = 1.1 ± 0.1 μm; CrISY: *k*_cat_ = 1.6 ± 0.1 s^−1^, *K_m_* = 4.5 ± 0.2 μm). Values are given as the mean ± S.D. of two independent measurements with different batches of protein. *d*, qRT-PCR shows tissue-dependent expression of ISY homologues in *A. majus*. Abundance of the Am29566 transcript was too low for quantification in all tissues. Expression values are given as the mean ± S.D. (four reactions). Each gene was separately normalized to the tissue with the highest expression level. Two replicates each were analyzed for two independent samples of cDNA.

Candidates for AmISY were identified based on sequence homology to ISY from *C. roseus* (CrISY) in a genome sequence of the JI7 inbred line of *A. majus* (http://snapdragon.genomics.org.cn/).^4^ The protein sequence of CrISY was used in a BLAST search against proteins predicted from the genome sequence to yield four hits with amino acid sequence identities between 39% and 66% (Fig. 2*a*). Candidate Am18679 showed the highest amino acid similarity to CrISY, with 66% identity and 79% similarity. For overexpression in *Escherichia coli*, all four genes were cloned from cDNA of *A. majus* flower and leaf tissue and successfully purified via nickel affinity chromatography. Enzyme reactions containing 8-oxogeranial and NADPH as substrates were analyzed by GC-MS. Only protein derived from candidate Am18679 (Fig. 2*b*), the candidate most similar to CrISY, yielded sizeable quantities of cyclized iridoid product (supplemental Fig. S1). Therefore, candidate Am18679 was named AmISY. Only trace amounts of substrate were consumed, and negligible products were detectable with the more distantly related candidates.

Efforts to directly test the physiological relevance of AmISY were not successful because silencing systems in *A. majus* using virus-induced gene silencing are highly inefficient (19, 20). However, our hypothesis that AmISY is the physiologically relevant iridoid synthase in *A. majus* is corroborated by the steady-state kinetic parameters (Fig. 2*c*; *k*_cat_ = 0.72 ± 0.02 s^−1^ and *K_m_* = 1.1 ± 0.1 μm), which are similar to those measured for CrISY, for which the physiological role has been confirmed by gene silencing (11). qRT-PCR of AmISY (Fig. 2*d*) with cDNA from *A majus* root, leaves, and flowers further indicated that AmISY is highly expressed in leaves and not expressed in roots and flowers. Although antirrhinoside is found in all *A. majus* tissues, the compound could be synthesized exclusively in leaves and then distributed throughout the plant. Phloem mobility of antirrhinoside has been demonstrated (2).

### Chiral analysis of the ISY reaction with model substrates

To investigate the stereoselectivity of the hydride transfer catalyzed by CrISY and AmISY, we initially analyzed reactions with model substrates lacking the 8-oxo group. These substrates can undergo enzymatic reduction, but the missing aldehyde moiety prevents subsequent cyclization (supplemental Fig. S2). With commercially available citral, a mixture of geranial (*E*-isomer) and neral (*Z*-isomer), as a substrate, CrISY yielded exclusively *S*-citronellal in a stereoconvergent fashion. In contrast, AmISY produced a 6:4 mix of *R*- and *S*-citronellal (supplemental Fig. S2*c*). To more closely reflect the structure of the physiological ISY substrate 8-oxogeranial, we synthesized geranial with low neral content (2.5%, supplemental Fig. S2*a*) by oxidation of geraniol with Dess-Martin periodinane. With this substrate, AmISY showed high stereoselectivity (supplemental Fig. S2*d*, 89% *R*). The *R* selectivity of AmISY observed here strongly supports the proposed biosynthesis of antirrhinoside via *R*-selective reduction of 8-oxogeranial. AmISY therefore appears to be the first example of an epi-iridoid synthase.

### Analysis of all ISY reaction products with 8-oxogeranial

**Figure 3.**
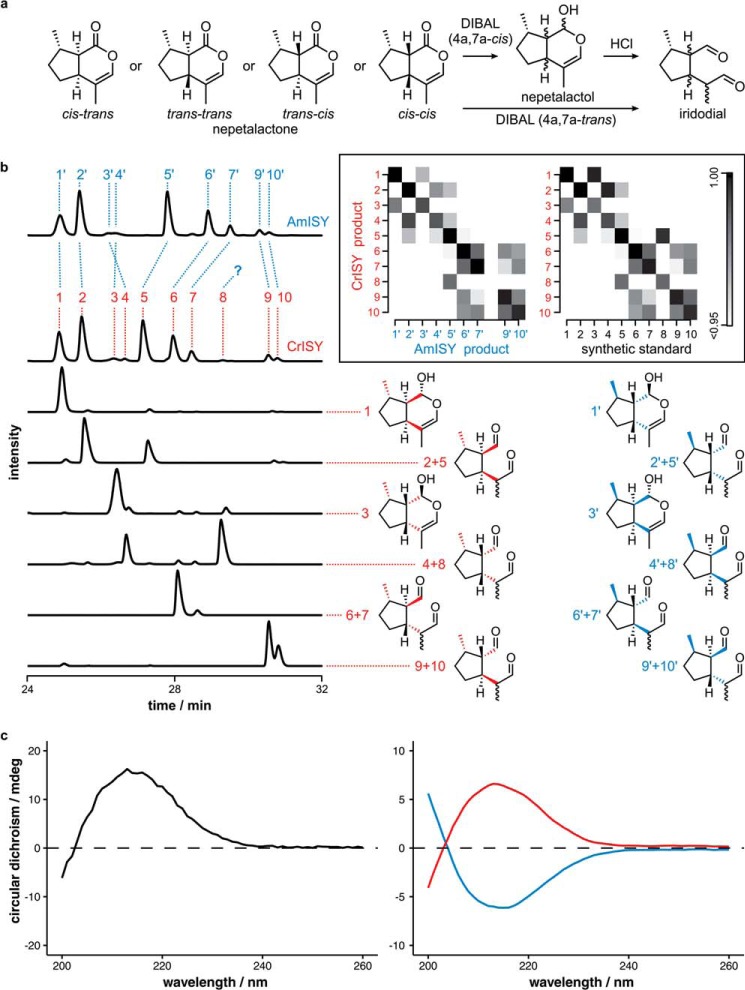
**Chiral GC-MS analysis of CrISY and AmISY products.** *a*, synthesis of nepetalactol and iridodial standards by DIBAL reduction of nepetalactones (11, 21, 24). *b*, reaction products of CrISY and AmISY with 8-oxogeranial substrate were analyzed on a chiral GC-MS column. The intensity axis of all chromatograms was normalized to the tallest peak. In the CrISY reaction, ten products could be identified (*red*) in comparison with authentic standards. AmISY products, which have the opposite chirality (*blue*), were matched to the CrISY products based on electron impact fragmentation spectra (supplemental Fig. S3). Product 8′ is presumably hidden under a larger peak in the AmISY chromatogram. The matching of CrISY and AmISY spectra (*left inset*) and the matching of CrISY and standard spectra (*right inset*) was verified by calculating pairwise similarity scores for all combinations, where a score of 1 signifies identity. The presence of similarity scores close to one on the diagonal confirms the peak assignment. *c*, circular dichroism spectra of 7*S-cis-trans* nepetalactol standard (*black*, 2 mm in hexane) and of the extracted CrISY (*red*) and AmISY (*blue*) reaction products from a reaction conducted in water without buffer. Water without buffer was used because buffer resulted in an attenuated CD signal.

To more rigorously assess the stereoselectivity of the two enzymes, AmISY and CrISY were assayed with the physiological substrate 8-oxogeranial. Analysis of this reaction is complicated by the fact that the product profile consists of a mixture of products. In *in vitro* assays, both nepetalactol and the open-form iridodials were observed, plus reduced, uncyclized product (Fig. 3). Additionally, small amounts of unidentified compounds were also observed. Before analysis of the stereoselectivity of the AmISY reaction with 8-oxogeranial, a method for resolving all components of the enzymatic reaction was developed, and all minor components of the ISY reaction were identified.

To do this, authentic standards of the side products needed to be prepared. Mass spectrometry data strongly suggested that the minor products of the CrISY reaction were alternative stereoisomers of nepetalactol and iridodial. Standards of *7S*-nepetalactol isomers can be obtained via diisobutylaluminum hydride (DIBAL-H) reduction of the *cis–cis*, *cis–trans*, *trans–cis*, and *trans—trans* nepetalactone isomers (Fig. 3*a*) (11, 21–23).^5^ However, the *trans–trans* isomer could not be isolated in sufficient quantities from plants and was instead generated by base-catalyzed isomerization of the *cis–cis* isomer, an inefficient uphill process with only 10% yield (21). Both nepetalactones with the ring fusion in *trans* configuration open directly to the corresponding iridodials (21) because of instability of the strained ring. Iridodials in *cis–cis* and *cis–trans* configuration were obtained by incubating the respective nepetalactols in 100 mm HCl overnight. The stereocenter at C1 in nepetalactol, which equilibrates in aqueous solution (21), and the stereocenter forming at C4 upon conversion to iridodial were not resolved. Each of these standards could be separated on achiral and chiral GC-MS columns.

With an analytical method and authentic standards for the *7S* stereoisomers in hand, the product profile of CrISY was assigned. In addition to *cis–trans* nepetalactols and *cis—trans* iridodials, the expected on-pathway intermediates for iridoid biosynthesis in *Catharanthus*, a number of other nepetalactol and iridodial diastereomers were observed. According to integrals of GC-MS peaks, combined *cis–trans*, *trans–trans*, *trans—cis*, and *cis–cis* species make up ∼69%, 21%, 5%, and 5% of the cyclized reaction products, respectively, under these *in vitro* assay conditions (Fig. 3*b*). Additionally, a substantial percentage of the entire product mix is reduced, non-cyclized *S*-8-oxocitronellal, as reported earlier (23%) (11).

### Chiral analysis of the AmISY and CrISY reaction with 8-oxogeranial

Having assigned all components of the enzymatic reaction, the spectra of AmISY and CrISY were compared. AmISY and CrISY reactions analyzed by GC-MS using a standard achiral column gave virtually identical chromatograms (supplemental Fig. S1). Chiral GC-MS, however, revealed substantial differences between the CrISY and AmISY product profiles (Fig. 3*b*).

Because enantiomers should have identical mass spectra, the diastereomers that were structurally identified in the GC-MS analysis of CrISY products could be matched to the corresponding AmISY enantiomers via the characteristic EI fragmentation spectra (Fig. 3*b* and supplemental Fig. S3). These spectra strongly suggest that CrISY and AmISY both generate a mixture of diastereomers but that the products of AmISY are exact mirror images of the CrISY products (Fig. 3*b*).

To further substantiate this hypothesis, CD spectra were obtained for the enzymatic product of CrISY and AmISY. As we predicted, the spectrum of the CrISY product showed an opposite sign compared with the AmISY spectrum, providing further support for the hypothesis that the AmISY product is enantiomeric to the CrISY product.

The analysis of the CrISY diastereomeric profile, as described above, revealed that the majority of the product forms the *cis–trans* isomer, or 4a*S*,7*S*,7a*R*. The stereochemistry of this isomer matches that of the downstream iridoid products in *C. roseus*. The major isomer found in the AmISY product profile must then correspond to 4a*R*,7*R*,7a*S* (*cis–trans*), which is the enantiomer of 4a*S*,7*S*,7a*R*. However, downstream *A. majus* iridoids are derived from the 4a*R*,7*S*,7a*R* isomer (*cis–cis*), which is found in only ∼5% of the cyclic AmISY products. Therefore, although AmISY generates the correct stereochemistry at the C7 position, the required *cis–cis* isomer is not the major product.

### Structural rationale for ISY stereoselectivity

To rationalize how AmISY generates the opposite stereocenter at C7, a homology model of AmISY was constructed. The homology model was calculated on the SwissModel server based on the CrISY structure in complex with geranic acid (PDB code 5DF1). There is a high level of amino acid similarity (79%) between AmISY and CrISY, so it is likely that the model accurately reflects the AmISY active site structure.

**Figure 4.**
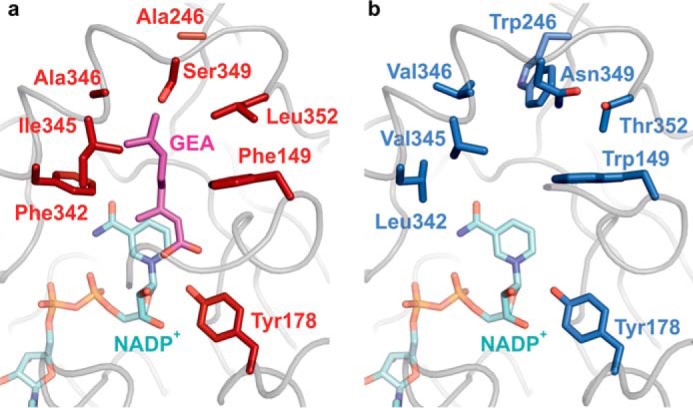
Shown are structural differences in the active sites of CrISY (*A*) and AmISY (*B*). A homology model of AmISY was constructed using the previously reported crystal structure of CrISY (PDB code 5DF1, Ref. 25) in complex with geranic acid (*GEA*, *pink sticks*) and NADP^+^ (*cyan sticks*). All binding pocket residues mutated in an attempt to invert the specificity of CrISY (supplemental Table S2) and the catalytic, conserved Tyr-178 are shown as *sticks*. The protein backbone is shown as a *light gray tube*.

The Lys-146 and Tyr-178 residues (Fig. 4) that are conserved in ISY homologues (25) and other short-chain dehydrogenases (26) are also present in AmISY. However, compared with CrISY, AmISY shows several large mutations in the 8-oxogeranial binding pocket, most notably A246W and F342L. Previously investigated iridoid synthases, OeISY from *Olea* (27), NISY from *Nepeta*,^5^ and *Catharanthus* homologues (28), resemble CrISY at these positions (supplemental Table S1), suggesting that these amino acids are at least partially responsible for the altered stereoselectivity in AmISY. However, we note that there are large phylogenetic distances covered by these enzymes.

In an attempt to graft the *R* selectivity of AmISY onto CrISY, we produced a series of CrISY mutants incorporating these sequence changes of AmISY (supplemental Table S2). In the construct CrISY-R1, two mutations, A246W and F342L, increased *R*-citronellal production from not detectable to 1%. Substitution of two additional residues (I345V and A346V) increased the fraction of *R*-citronellal 17-fold. Another mutation added to CrISY-R1 (F149W) achieved a 7-fold increase in *R* product. Either way, the high stereoselectivity of AmISY was not attained. Additional sequence changes, perhaps at second-shell residues, must be required to guarantee selective hydride transfer.

## Discussion

Epi-iridoids are pervasive in the Plantaginaceae as well as numerous other plant families (such as Lamiaceae (29), Rubiaceae (30), Orobanchaceae (31), and Paulowniaceae (29)). With several hundred epi-iridoid–derived structures reported, AmISY may become a reference point for the identification of epi-ISY enzymes in these pathways. We predict that these epi-synthases can be identified by inspection of the distinct active site residues (Trp-149, Trp-246, Leu-342, Val-345, and Val-346) identified by the AmISY homology model and by mutation.

The generation of the *S* configuration at C7 is well explained by the preference of CrISY to transfer a hydride from the pro-*S* face to 8-oxogeranial, as demonstrated in biochemical assays (11), and can be structurally rationalized by the crystal structure of the enzyme bound to a geranic acid inhibitor (25). We hypothesize that the active site residues of AmISY are at least partially responsible for binding the 8-oxogeranial substrate in the opposite orientation (Fig. 4), thereby changing the stereochemistry of hydride addition to generate the *R* stereocenter. The newly discovered *R*-selective cyclization by AmISY may offer valuable biocatalytic access to a larger range of poorly accessible iridodial- and nepetalactol-related synthons (32).

Enzymes typically exert tight control over the course of a reaction by embedding the transition states and intermediates in a precisely tailored binding pocket that allows no other than the desired orientation of the reactive groups. However, in a few notable cases, reactions in nature are (partially) uncatalyzed (33). For example, in certain cationic cyclizations of terpenes, the role of cyclases has been argued to be limited to generation and protection of the cationic intermediates without full control of the cyclization process (34). Given the mixture of products that result from CrISY and AmISY under *in vitro* reaction conditions, the cyclization half-reaction of iridoid synthase may be another such example of an uncatalyzed reaction. We hypothesize that the high selectivity of the reduction step and the relatively poor selectivity of the cyclization step in iridoid synthases are mechanistically best explained by enzymatic, stereoselective reduction followed by cyclization in an achiral environment outside the active site.

Two observations support this scenario. First, the cyclization step is not only relatively poorly controlled, but the product ratio is also insensitive to mutations with large impact on the overall NADPH consumption rate (see the supplemental information of Ref. 25). Second, the enantiomeric enolate intermediates produced by CrISY and AmISY (Fig. 1) constitute a sensitive probe for the environment in which cyclization happens. Any selectivity imposed on the cyclization by a chiral protein binding pocket would be highly unlikely to affect these enantiomeric enolates and the transition states for the observed product range in the same fashion. However, the products of both CrISY and AmISY appear to be exact mirror images, as shown by the superimposable achiral GC-MS chromatograms and CD signals of opposite sign.

The metabolic role of AmISY in the plant is supported by the high expression levels in leaves, the physiologically relevant catalytic parameters, and the *R* selectivity that is consistent with earlier feeding experiments. Moreover, no other iridoid synthase homologs from *A. majus* were highly active *in vitro*. However, if we make the reasonable assumption that AmISY is the metabolically relevant enzyme, then the mechanism by which the diverse AmISY products are channeled into the pathway of the abundant natural product antirrhinoside remains an unsolved problem. Biosynthesis of antirrhinoside requires the C7-*R* stereochemistry, which is indeed produced by AmISY. However, antirrhinoside also requires the *cis—cis* diastereomer. In contrast, the major product of AmISY is C7-*R-cis-trans*, whereas C7-*R-cis-cis* makes up only a few percent of the product.

It is likely that additional enzymes are required to isomerize the major AmISY product so that it can be diverted into the antirrhinoside biosynthetic pathway. For example, the *trans—trans* iridodial could be converted into the more stable *cis—cis* iridodial by an epimerase that abstracts the labile C7a-H proton next to the C1-carbonyl (Fig. 1). Alternatively, iridoid synthase could utilize a helper protein to control the stereochemistry of cyclization. This question is not unique to the antirrhinoside pathway. In *Nepeta* species, a variety of nepetalactones with varying stereochemistry at the C4a and C7a carbons are observed. A species of *Nepeta mussinii* that exclusively produces the *trans–cis* iridoid isomer as a final product has an iridoid synthase that predominantly produces the *cis–trans* isomer. This mismatch also strongly suggests that additional enzymes are required to set the stereochemistry at the iridoid bridgehead carbons in *Nepeta*.^5^

Iridoids show a vast array of stereochemical variation in their core scaffold. This stereochemical variation is essential for the structural diversity and range of biological activities found in this class of compounds. How iridoid stereochemistry is controlled during the course of biosynthesis still remains cryptic. However, the discovery of AmISY clearly demonstrates that an alternative class of iridoid synthase is responsible for setting the stereochemistry of C7 by controlling the reduction of the 8-oxogeranial substrate. The discovery of AmISY provides insight into how nature controls the stereochemistry of this important class of compounds.

## Experimental procedures

### Cloning

RNA was isolated from *A. majus* tissue and purified using the RNeasy Plant Minikit (Qiagen) before reverse transcription using the SuperScript III reverse transcriptase kit (Thermo Fisher Scientific), following the protocols of the suppliers. The candidate genes were PCR-amplified from the cDNA using gene-specific oligonucleotides (supplemental Table S3). In a second PCR reaction, the fragments were endowed with complementary overhangs for subsequent InFusion cloning (Clontech Laboratories) into the pOPINF expression vector (Addgene, 26042) (35).

### Protein production

The candidates were expressed in N-terminally His-tagged form using soluBL21 (DE3) (Genlantis) cells transformed with pOPINF plasmids carrying the desired construct and purified similar to a procedure published previously (25). A flask containing 1 liter of yeast extract and Tryptone medium and 50 μg/ml carbenicillin was inoculated with 1 ml of overnight culture of the expression strain and incubated at 37 °C until the *A*_600_ reached 0.6–0.8. The temperature was then reduced to 18 °C, protein production was induced by adding 0.25 mm isopropyl 1-thio-β-d-galactopyranoside, and incubation was continued for 16–20 h. Cells were harvested by centrifugation at 4000 × *g* for 20 min at 4 °C and resuspended in 50 ml of buffer A (50 mm Tris-HCl, pH 8.0, 50 mm glycine, 5% v/v glycerol, 500 mm NaCl, 20 mm imidazole, and 1 mm β-mercaptoethanol) containing 0.5 mg/ml lysozyme and one tablet of Complete EDTA free protease inhibitor (Roche). Cells were disrupted by sonication on ice for 7 min (2-s sonication, 3-s break). Cell debris was removed from the lysate by centrifugation at 35,000 × *g* for 20 min at 4 °C. The supernatant was injected on a His-Trap 5-ml nickel affinity column attached to an Äkta purifier (GE Healthcare). Protein was eluted with buffer A containing 500 mm imidazole. Iridoid synthase–containing fractions were pooled, concentrated, and washed with size exclusion buffer B (20 mm HEPES, pH 7.5, 150 mm NaCl, and 1 mm β-mercaptoethanol) in an Amicon centrifugal filter (Millipore) with 30-kDa molecular mass cutoff. For further purification, the protein was loaded onto a HiLoad 16/600 Superdex 200 pg (GE Healthcare) size exclusion column and eluted with buffer B. Protein concentration was determined in triplicate on a Nanodrop spectrophotometer (Thermo Fisher Scientific) using absorbance at 280 nm and calculated extinction coefficients (ExPASy ProtParam; Am18679, 99,350 m^−1^ cm^−1^; Am18685, 93,390 m^−1^ cm^−1^; Am26155, 101,870 m^−1^ cm^−1^; Am29566, 81,360 m^−1^ cm^−1^). Protein was flash-frozen in liquid nitrogen and stored at −20 °C until further assays were performed.

### NADPH consumption assay

The catalytic parameters *k*_cat_ and *K_m_* of AmISY were determined by spectrophotometrically measuring the initial rate of NADPH consumption at 340 nm and 25 °C on a Lambda35 (PerkinElmer Life Sciences) spectrophotometer. Reactions were conducted in plastic cuvettes with 1-cm path length and contained 20 nm AmISY in buffer C (200 mm MOPS, pH 7.0, and 100 mm NaCl), 50 μm NADPH (Sigma, N7505), 0.66–5 μm 8-oxogeranial substrate, and 1% THF in a total volume of 800 μl. The substrate 8-oxogeranial was synthesized as described previously from geranyl acetate (11), stored as a 50 mm stock solution in inhibitor-free tetrahydrofuran at −80 °C, and diluted to the appropriate concentration in water. Reactions were started by addition of enzyme. The background rate before addition of enzyme (2.52 10^−6^ optical density/s) was subtracted, and initial velocities were calculated using the extinction coefficient of NADPH (6220 m^−1^ cm^−1^). Catalytic parameters were calculated in Kaleidagraph 4.0 by nonlinearly fitting a plot of the initial velocities *versus* substrate concentration to the Michaelis–Menten equation.

### qRT-PCR

To quantify expression levels, RNA was isolated from samples of leaf, flower, and root tissue of two *A. majus* plants using the RNeasy Plant Minikit (Qiagen). cDNA was prepared from 1 μg of total RNA using the iScript cDNA synthesis kit (Bio-Rad). qRT-PCR was performed on a CFX96 real-time PCR detection system (Bio-Rad) using SSO Advanced SYBR Green Supermix (Bio-Rad). For each of the four candidates, gene-specific oligonucleotides were designed (supplemental Table S3) to amplify a 100-bp long section of the open reading frame, and their individual efficiency was tested. For comparative analysis of the expression of each gene in leaf, flower, and root, the detected transcript levels were compared with the tissue with the highest expression level using the Δ C_T_ method (36).

### GC-MS analysis

A protocol for achiral GC-MS analysis of ISY reactions was adapted from procedures published previously (11, 25). Reactions were conducted in a total volume of 50 μl of buffer C containing 0.5 mg/ml nickel affinity-purified enzyme, 0.8 mm NADPH, and 0.6 mm 8-oxogeranial. After 30 min at 30 °C, products were extracted with 100 μl of ethyl acetate in a 400-μl flat-bottom glass insert (Agilent, 5181-3377) in a GC-MS vial closed with a polytetrafluoroethylene septum. Phase separation was improved by centrifugation of the glass insert in a 2-ml plastic tube at 2000 × *g* for 2 min. A volume of 3 μl of the clear supernatant was injected in splitless mode on a Hewlett Packard 6890 GC-MS equipped with an Agilent HP-5MS 5% phenylmethylsiloxane column (30 m × 250 μm, 0.25-μm film thickness), a 5973 mass selective detector, and an Agilent 7683B series injector and autosampler. The front inlet temperature was set to 220 °C. After an initial hold at 60 °C for 5 min, a thermal gradient was run from 60° to 150 °C at 20 K/min, from 150 °C to 280 °C at 45 K/min, with a final hold of 4 min at a helium flow rate of 37 cm/s and 1 ml/min. After a solvent delay of 5 min, electron impact fragmentation spectra from 50–300 *m*/*z* were collected at a fragmentation energy of 70 eV.

This protocol was adapted for chiral analysis of citral and geranial reduction. Reactions were run for 180 min at 30 °C and contained 0.5 μm enzyme, 1 mm citral (Sigma-Aldrich, C83007) or geranial, 2% tetrahydrofuran as a co-solvent, 1 mm NADPH, and buffer C up to a volume of 300 μl. Commercial *rac*-citronellal (Sigma-Aldrich, 27470) and *S*-citronellal (TCI, C1454) were used as standards. From 100 μl of ethyl acetate extract, 1 μl was injected at a 10-fold split ratio into a Restek SKY Liner with wool for split injection. The chiral separation was performed on a Supelco β-DEX225 column (30 m × 250 μm, 0.25-μm film thickness) with an isothermal gradient at 93 °C for 33 min at an average velocity of 26 cm/s. Runs were concluded with a temperature gradient up to 220 °C at a rate of 40 K/min and a final hold time of 4 min.

Further modifications were made to the analytical protocol to allow separation of the products of 8-oxogeranial cyclization. Concentrations of 8-oxogeranial and NADPH in the enzyme reaction were set to 0.5 and 1 mm, respectively. The injection volume was set to 1 μl and the split ratio at the GC-MS injector to 6-fold, and, after an initial hold of 5 min at 105 °C, a thermal gradient was run from 105–150 °C at a rate of 1.5 K/min and from 150–220 °C at 60 K/min with a final hold of 4 min. For quantitative comparison of spectra, they were integrated across the entire peak, and background was subtracted in AMDIS-32. Similarity was calculated with the SpectrumSimilarity function of the OrgMassSpecR package in R version 3.3.3 as the cosine of the angle between the intensity vectors.

### CD spectroscopy

Enzyme reactions for CD spectroscopy were conducted for 5 h at 30 °C in water with 0.4 mm 8-oxogeranial and 1 mm NADPH as substrates and 0.5 μm enzyme. Enzyme was diluted at least 150-fold from a buffered solution. Products were extracted with ethyl acetate. The extract from a 1.6-ml reaction was evaporated and taken up in 200 μl of hexane. Completeness of the reaction was verified by GC-MS. Spectra were recorded in 1-nm steps with 0.5-s averaging time on a Chirascan Plus spectropolarimeter (Applied Photophysics) at 20 °C in a 1-mm cuvette. Three measurements were averaged, and background with only hexane was subtracted.

### Chemicals

All compounds except *trans—trans* iridodial have been described previously (11, 21).^5^ The identity and purity of compounds were verified based on NMR spectra recorded on a Bruker 400-MHz/54-mm UltraShield Plus long hold time automated spectrometer at 400 MHz (^1^H NMR) and 100 MHz (^13^C NMR). The residual solvent peak of chloroform was adjusted to δ 7.26 (^1^H NMR) and 77.16 (^13^C NMR). Assignment of peaks was aided by two-dimensional ^1^H-COSY and ^1^H-^13^C-HSQC data (supplemental information).

Geranial with a low level of neral contamination was synthesized by oxidizing 100 mg of geraniol (0.65 mmol, 1 eq) in a suspension of 327 mg of sodium bicarbonate (3.9 mmol, 6 eq) in 40 ml of dichloromethane with 330 mg of Dess-Martin periodinane (0.78 mmol, 1.2 eq). The reaction was stirred on ice for 90 min and worked up by filtration over a 0.5-cm glass column packed with 6 cm of silica gel on top of 1 cm of anhydrous sodium bicarbonate. The column was washed with 50 ml of 50% diethylether in hexane, and the product was eluted with 100 ml of diethylether. The solvent was evaporated to dryness, the residue was taken up in 4 ml of hexane and filtered over PTFE, and the solvent was evaporated to yield 74 mg of clear oil (0.49 mmol). The product was identified as geranial based on GC-MS analysis and comparison with the National Institute of Standards and Technology (NIST) library and commercial citral (supplemental Fig. S2*a*).

*Trans–cis* nepetalactone was isolated from catnip oil by silica flash chromatography as described by Sherden *et al.*^5^ *Cis—trans* nepetalactone synthesis from the same product via base-catalyzed isomerization has been described by Geu-Flores *et al.* (11). Reduction to the corresponding *trans—cis* nepetalactol and *cis—trans* iridodial has been described previously (11).^5^

*Cis–cis* nepetalactone was isolated from a *Nepeta* variety. To identify a plant containing the correct isomer, *Nepeta* plants were obtained from plant nurseries (Crocus Ltd., Windlesham, UK; Burncoose Nurseries, Gwennap, Cornwall, UK; Herbal Haven, Saffron Walden, UK; Hardy's Cottage Garden Plants, Hampshire, UK). For methanol extraction, 30–50 mg of fresh leaves were frozen in liquid nitrogen and ground to fine powder in 2-ml Safe-Seal plastic tubes with tungsten beads in a ball mill. After addition of 300 μl of methanol to the cold tube, the tube was vortexed. The resulting slurry was transferred to a 2-ml glass vial with a screw cap, and 600 μl of HPLC-grade hexane was added. After vortexing for 10 s, a green hexane layer on top separated from a lighter yellow methanol layer with bleached particles. The hexane phase containing nepetalactones was transferred to a solid-phase extraction column (Phenomenex Strata SI-1 Silica, 55 μm, 70 Å, 100 mg/1 ml) with a Pasteur pipette. Nepetalactones were eluted with 500 μl of 20% ethyl acetate in hexane. For identification of diastereomers, a volume of 2 μl was injected in split mode (50-fold) on the GC-MS instrument described above. Separation was performed on a Phenomenex Zebron ZB5-HT column (5% polyphenylmethylsiloxane; length, 30 m; diameter, 250 μm; film thickness, 0.10 μm) with a 5-m guard column. Helium was used as mobile phase at a constant flow rate of 7.4 ml/min and average velocity of 100 cm/s. After 5 min at 80 °C, the column temperature was increased to 110 °C at a rate of 2.5 K/min, then to 280 °C at 120 K/min, and kept at 280 °C for 4 min. Nepetalactones eluted in the sequence *trans–trans* (14.16 min), *cis–trans* (14.48 min), *trans–cis* (15.71 min), and *cis–cis* (15.99 min).

To isolate preparative quantities of *cis—cis* nepetalactone, all green from a flowerless *N. mussinii* 'Snowflake' plant (Burncoose Nurseries) was cut off a few centimeters above the soil (approximately 40 g wet weight). The tissue was thoroughly blended in a kitchen blender together with 160 ml of water. Water was added up to approximately 500 ml, and organic compounds were extracted with 5 × 100 ml of dichloromethane. The combined fractions were filtered over paper and washed with 200 ml of brine in a separation funnel. The organic phase was dried by adding anhydrous sodium sulfate, and the solvent was evaporated under reduced pressure. The solid residue was taken up in 10 ml of hexane and separated by silica flash chromatography on a 3 × 25 cm column packed in hexane. Compounds were eluted with a gradient from 10–20% ethyl acetate in hexane in steps of 2% (200 ml each). Elution fractions were checked for diastereomeric purity by GC-MS (see above), and pure fractions were pooled and evaporated, yielding 120 mg of yellow oil. The compound was identified as *cis—cis* nepetalactone in comparison with published ^1^H NMR spectra (21).

*Cis—cis* nepetalactol ([4a*R*,7*S*,7a*S*]-4,7-dimethyl-1,4a,5,6,7,7a-hexahydrocyclopenta[*c*]pyran-1-ol) was obtained by reducing 92 mg (0.55 mmol, 1 eq) of c*is–cis-*nepetalactone with 95 mg of DIBAL (0.66 mmol, 1.2 eq). Under dry conditions and a nitrogen atmosphere, 710 μl of DIBAL dissolved in hexane was added dropwise during 20 min to a dry ice/acetone–cooled flask containing *cis—cis* nepetalactone in 5 ml of hexane while stirring. After stirring for another hour, 770 mg of Bäckstrøm reagent (sodium sulfate decahydrate:celite, 1:1, v/v) was added, and the reaction was stirred for another hour on ice. Solid particles were removed by filtration on a glass frit, which was washed with diethyl ether. The residue obtained after removal of solvent under reduced pressure was purified by silica flash chromatography (1.5 × 21 cm column, eluted with up to 20% ethyl acetate in hexane), yielding 35 mg of product (0.21 mmol, 38% yield) as a 70:30 mix of C1 anomers according to NMR. ^1^H NMR (major anomer): δ 6.02 (1H, dq, *J* = 1.4/1.4 Hz, C3), 5.01 (1H, dd, *J* = 6.2/5.4 Hz, C1), 2.72 (1H, d, *J* = 6.3 Hz, O1), 2.47 (1H, broad ddd, J = 7/7/7 Hz, C4a), 2.26–2.20 (1H, m, C7), 2.16–2.08 (1H, m, C7a), 1.87–1.79 (1H, m, C5), 1.82–1.72 (1H, m, C6), 1.59–1.51 (1H, m, C5), 1.55 (3H, dd, *J* = 1.1/1.4 Hz, C8), 1.35–1.25 (1H, m, C6), 1.09 (3H, d, *J* = 7.1 Hz, C9); ^13^C NMR (major anomer): δ 134.58 (C3), 113.81 (C4), 92.85 (C1), 45.53 (C7a), 38.51 (C4a), 36.01 (C7), 32.54 (C6), 29.70 (C5), 16.73 (C9), 16.47 (C8); ^1^H NMR (minor anomer): δ 5.98 (1H, dq, *J* = 1.5/1.5 Hz, C3), 5.33 (1H, dd, *J* = 5.0/3.3 Hz, C1), 2.52 (1H, dd, *J* = 5.0/1.3 Hz, O1), 2.38–2.20 (1H, m, C7), 2.36–2.29 (1H, m, C4a), 2.20–2.12 (1H, m, C7a), 2.19–2.10 (1H, m, C5), 1.64–1.53 (1H, m, C5), 1.60 (3H, dd, *J* = 0.9/1.4 Hz, C8), 1.15 (3H, d, *J* = 7.0, C9), C6 is not assigned; ^13^C NMR (minor anomer): δ 132.55 (C3), 92.75 (C1), 44.46 (C7a), 38.99 (C4a), 36.71 (C7), 30.97 (C5), 17.24 (C8), 15.29 (C9), C4 and C6 are not assigned.

*Trans—trans* nepetalactone was not found in any *Nepeta* plant in sufficient quantities and had to be synthesized by epimerization of *cis—cis* nepetalactone under basic conditions (21). In a 50-ml flask equipped with a reflux condenser, 500 mg of *cis—cis* nepetalactone (1 eq, 3.0 mmol) was dissolved in toluene and refluxed. Progress of the reaction was controlled by GC-MS, and the reaction was stopped when the equilibrium was reached at a 9:1 *cis–cis*:*trans–trans* ratio after 6 h. The reaction mix was evaporated under reduced pressure and separated by silica flash chromatography as described for *cis—cis* nepetalactone. Fractions were checked for the nepetalactone diastereomers by TLC (anisaldehyde stain, 20% ethyl acetate in hexane as eluent), where *trans–trans* (R_f_ = 0.65) and *cis–cis* nepetalactone (R_f_ = 0.59) were well separated. The ^1^H NMR was identical to a spectrum published previously (21).

*Trans—trans* nepetalactone was reduced to *trans—trans* iridodial ([1*R*,2*S*,5*S*]-2-methyl-5-[1-oxopropan-2-yl]cyclopentane-1-carbaldehyde) following the procedure described for the *cis—cis* isomer. Only the major stereoisomer at C7 (approximately 90%) could be assigned. ^1^H NMR: δ 9.63 (1H, d, *J* = 2.1 Hz, C6), 9.59 (1H, d, *J* = 3.6 Hz, C9), 2.59 (1H, dddd, all *J*≈8 Hz, C5), 2.40 (1H, ddq, *J* = 2.1/7.3/7.3 Hz, C7), 2.18–2.13 (1H, m, C2), 2.16–2.08 (1H, m, C1), 1.99–1.91 (1H, m, C4), 1.95–1.86 (1H, m, C3), 1.57–1.49 (1H, m, C4), 1.39–1.33 (1H, m, C3), 1.082 (3H, d, *J* = 7.2 Hz, C8), 1.078 (3H, d, *J* = 6.4 Hz, C10). ^13^C-NMR: δ 204.08 (C6 or C9), 203.46 (C6 or C9), 63.48 (C1), 50.86 (C7), 41.26 (C5), 37.18 (C2), 34.00 (C3), 29.58 (C4), 19.34 (C10), 12.37 (C8). Acid opening of *cis–cis* and *cis—trans* nepetalactol was achieved by treatment with 100 mm hydrochloric acid as described for *cis—trans* nepetalactol.^5^

## Author contributions

H. K. and S. E. O. conceived the study. H. K. and F. K. cloned ISY genes. H. K. and M. O. K. prepared standards. H. K., F. K., and M. O. K. performed biochemical assays. H. K. and S. E. O. wrote the manuscript.

## Supplementary Material

Supplemental Data

## Footnotes

4 Please note that the JBC is not responsible for the long-term archiving and maintenance of this site or any other third party–hosted site.

5 N. H. Sherden, B. R. Lichman, L. Caputi, D. Zhao, M. O. Kamileen, C. R. Buell, and S. E. O'Connor, submitted for publication.

## Footnotes

3 The abbreviations used are:

ISY: iridoid synthase
AmISY: *Antirrhinum majus* iridoid synthase
CrISY: *Catharanthus roseus* iridoid synthase
qRT-PCR: quantitative real-time PCR
DIBAL: diisobutylaluminum
HSQC: heteronuclear single quantum coherence spectrum.
