# Supplementary material for: Inverted stereocontrol of iridoid synthase in snapdragon
Source: J Biol Chem. 2017 Jul 12;292(35):14659–67. doi: 10.1074/jbc.M117.800979 (PMC5582856; doi:10.1074/jbc.M117.800979)
Supplement: Supplemental Data [file supp_292_35_14659__index.html]

Inverted stereocontrol of iridoid synthase in snapdragon — Inverted stereocontrol of iridoid synthase in snapdragon — Discovery of epi-iridoid synthase — Supplemental Data 

# Inverted stereocontrol of iridoid synthase in snapdragon

## Supplemental Data

- Supplemental Data (.pdf, 1.0 MB) - All Supplemental Tables and Figures
