# Supplementary material for: Inverted stereocontrol of iridoid synthase in snapdragon
Source: J Biol Chem. 2017 Jul 12;292(35):14659–67. doi: 10.1074/jbc.M117.800979 (PMC5582856; doi:10.1074/jbc.M117.800979)

# INVERTED STEREOCONTROL OF IRIDOID SYNTHASE IN SNAPDRAGON

**Hajo Kries[1,2], Franziska Kellner[1], Mohamed Omar Kamileen[1], Sarah E. O'Connor[1,3]**
*[1]Department of Biological Chemistry, The John Innes Centre, Norwich, NR4 7UH, UK [2]Current address: Leibniz Institute for Natural Product Research and Infection Biology e.V. (Hans-Knöll-Institute), 07745 Jena, Germany*

[3] To whom correspondence should be addressed: Sarah E. O'Connor, Dept. of Biological Chemistry, The John Innes Centre Norwich UK Fax: 44 (0)1603 450018, E-mail: sarah.oconnor@jic.ac.uk.

# Supplemental Data

**Supplemental Tables S1-S3**

**Supplemental Figures S1-S3**

**Supplemental NMR Data**

## Supplementary Tables

TABLE S1. **Comparison of ISY binding pockets.***

| | % ID | Type | 149 | 246 | 342 | 345 | 346 | 349 | 352 |
|---|---|---|---|---|---|---|---|---|---|
| **CrISY** | 100 | ISY | F | A | F | I | A | S | L |
| **OeISY** | 72.3 | ISY | I | V | F | I | A | L | V |
| **NcISY2** | 54.5 | ISY | V | S | F | T | I | N | R |
| **AmISY** | 65.6 | epiISY | W | W | L | V | V | N | T |

*Positions relative to CrISY. Cr: *Catharanthus roseus* (periwinkle, genbank JX974564), Oe: *Olea europaea* (olive, genbank KT954038), Nc: *Nepeta cataria* (catnip, genbank KY882234), Am: *Antirrhinum majus* (snapdragon, genbank Am.18679)

TABLE S2. **Grafting the binding pocket of AmISY on CrISY.***

| construct | mutations | ***R*-citronellal (%)** |
|---|---|---|
| CrISY | - | n.d. |
| CrISY-R1 | A246W, F342L | 0.97 |
| | **CrISY-R1 plus:** | |
| CrISY-R4 | F149W | 7.27 |
| CrISY-R5 | S349N, L352T | 1.32 |
| CrISY-R6 | I345V, A346V | 17.08 |
| CrISY-R7 | F149W, I345V, A346V, S349N, L352T | 3.42 |
| AmISY | - | 88.90 |

*The fraction of *R*-citronellal is given for reactions with (2*E*)-geranial. Residue numbering is relative to CrISY. N.d.: not detectable.

TABLE S3. **Oligonucleotides for cloning and qRT-PCR.**

| **Name** | **Sequence (5' to 3')** |
|---|---|
| 55_f | ATGAGCTGGTGGTGGGC |
| 55_r | TCAAGGAACTATCTTGTAAGCCTTCACT |
| 66_f | ATGGGCTCCATTGATGC |
| 66_r | TCATGGGATAAATTTCTCGGCTC |
| 79_f | ATGAGCTGGTGGTATAGAAGA |
| 79_r | TTAAGGAATAAACTTGAAATCTCTCATTTTGTTAATTG |
| 85_f | ATGGCGAGCTGGTGGTGG |
| 85_r | TCATGGAACAATTTTGTGAGC |
| qRT_55_f | CCGTTTAGCATGATGAATTTGGTG |
| qRT_55_r | GCCTTAGAACCTGGAAACCT |
| qRT_66_f | TCATAATGGGTTGTTCAAAGAAAACG |
| qRT_66_r | CCAAACACGAAAGGGAGATTCA |
| qRT_79_f | CCTTGTAGTATGATGAACACTGTCA |
| qRT_79_r | GACGTTTCAGTTCCGGTATACAC |
| qRT_85_f | TAGCATGATGAACATGATGGACTC |
| qRT_85_r | TAGCAGTTCCAGGAGGCT |

## Supplementary Figures

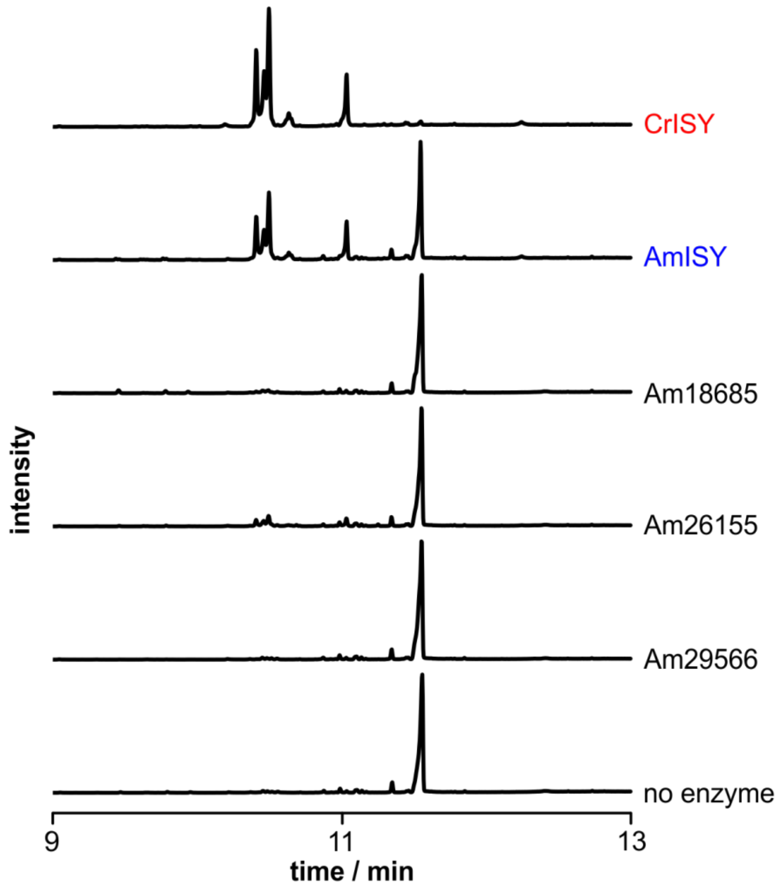


FIGURE S1. **GC-MS of AmISY candidates compared to CrISY.** Although conversion is not complete, candidate Am18679 (AmISY) is the only enzyme showing a product profile similar to CrISY. The other candidates generate very little (Am26155) or no product.

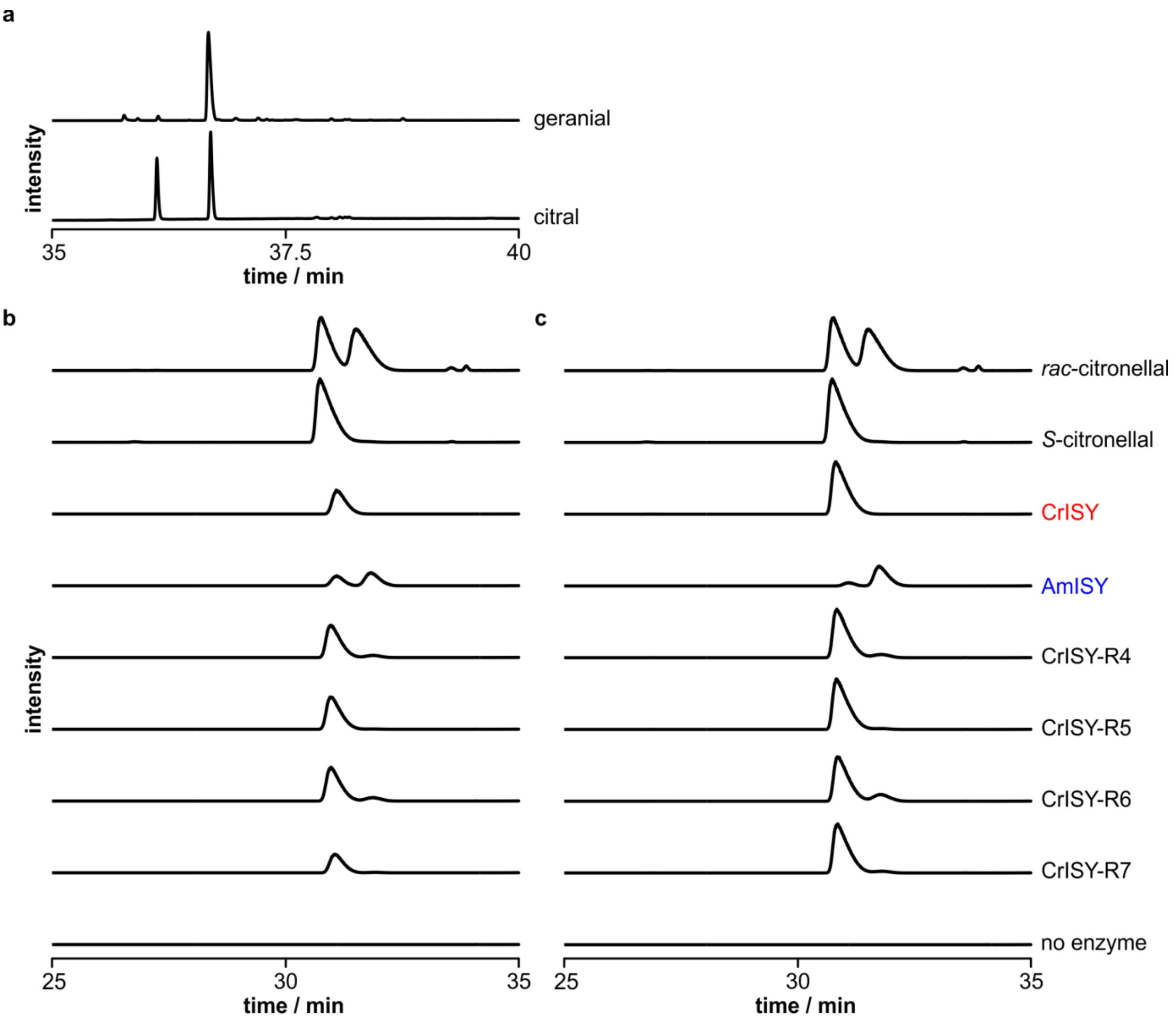


FIGURE S2. **GC-MS analysis of citral and geranial conversion.** a) In comparison to commercial citral (bottom), which is a mixture of geranial and neral, geranial ($t_R$= 36.675 min) obtained by careful oxidation of geraniol contains smaller quantities of the *2Z* configured neral isomer (2.5%, $t_R$= 36.138 min). Citral (a) and geranial (b) were reduced to citronellal with CrISY, AmISY and CrISY mutants R1-R4. a) Enzymatic conversion of citral shows stereoconvergence with CrISY (100% *S*-citronellal) and a mixture of *R*- and *S*-citronellal with AmISY (61% *R*- and 39% *S*-citronellal). b) Geranial afforded similar product profiles with most enzymes but with AmISY, the fraction of *R*-citronellal was increased to 89%. Among the CrISY-R mutants, the highest fraction of *R*-citronellal was obtained with CrISY-R6 (17% *R*-citronellal; A246W, F342L, I345V, A346V). Chromatograms in a) are scaled to the largest peak, whereas chromatograms in b) and c) are all scaled to the same intensity.

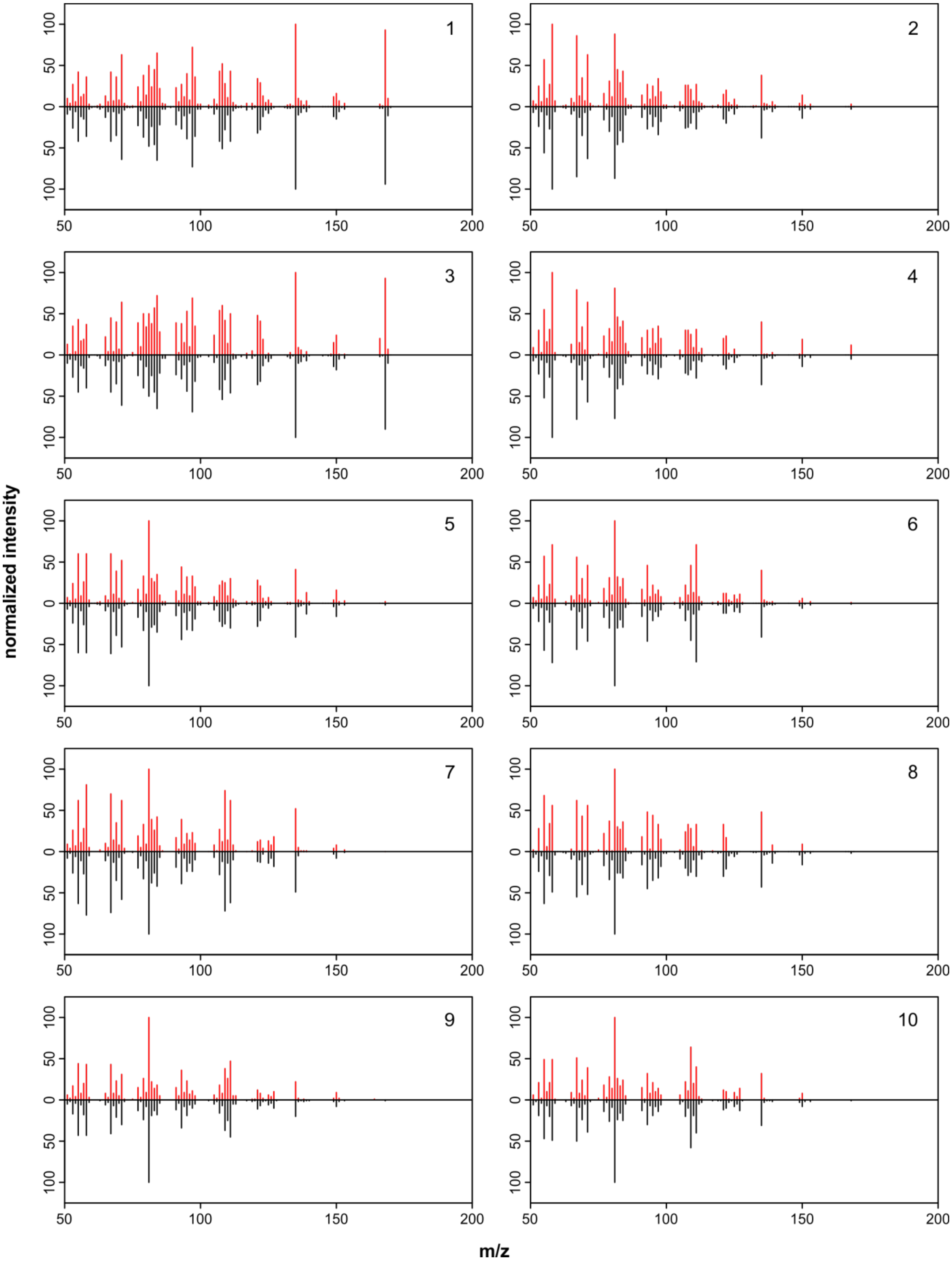


(continued on next page)

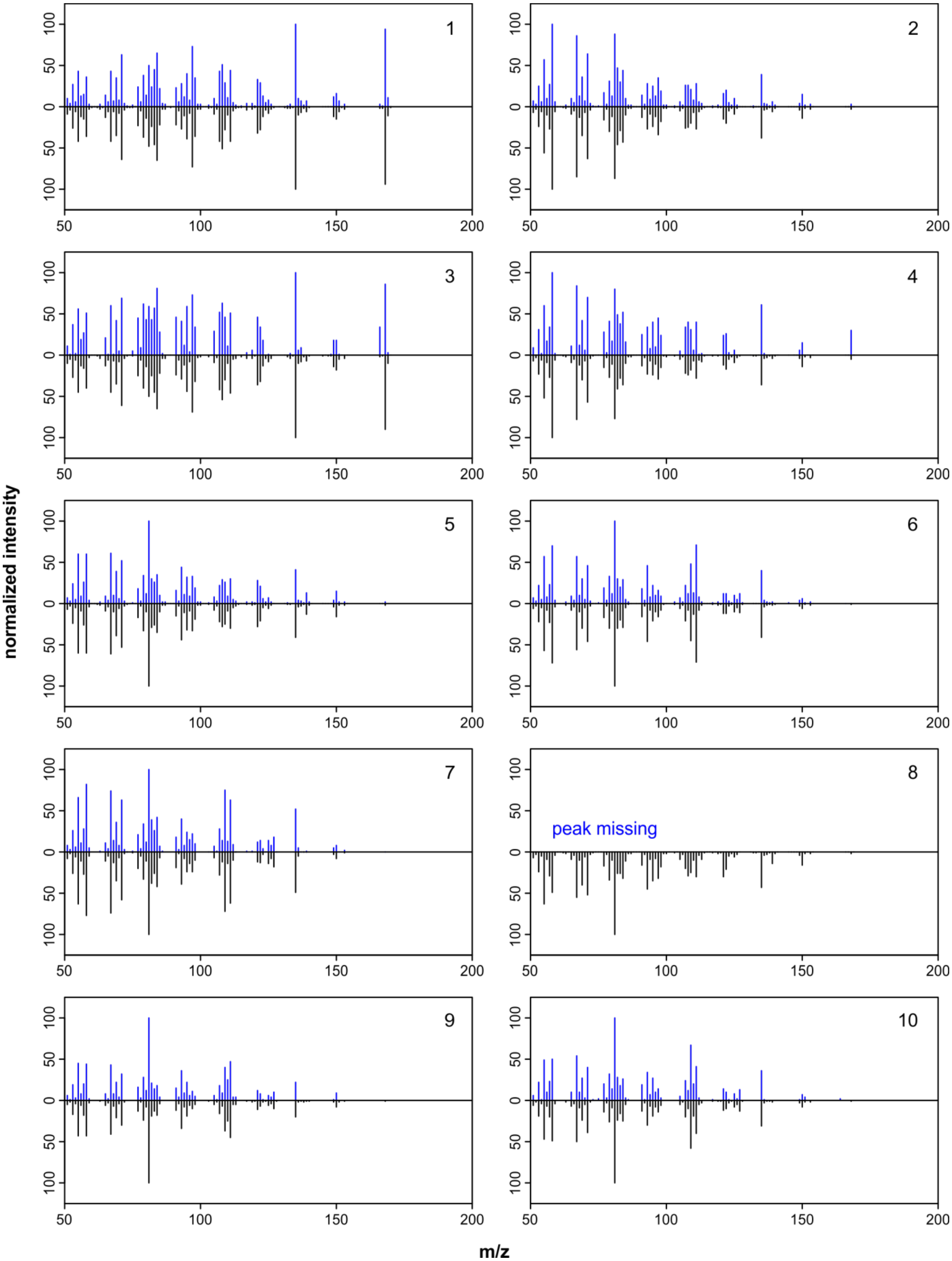


FIGURE S3. **GC-MS spectra of CrISY (red) and AmISY (blue) products compared to authentic standards (black).** Peak numbering (upper right corner) refers to **Fig. 3**.Spectra were calculated in AMDIS32 by manually integrating all scans belonging to one peak and subtracting

background signal before or after the peak. Compound 8’ is missing in the AmISY chromatogram. Since AmISY products are enantiomers of the CrISY products, retention times on the chiral column (**Fig. 3**) are different, but the spectra are identical to those of the standards with opposite chirality.

# NMR spectra

*Cis*-*cis*-nepetalactone $^1$H-NMR in $CDCl_3$

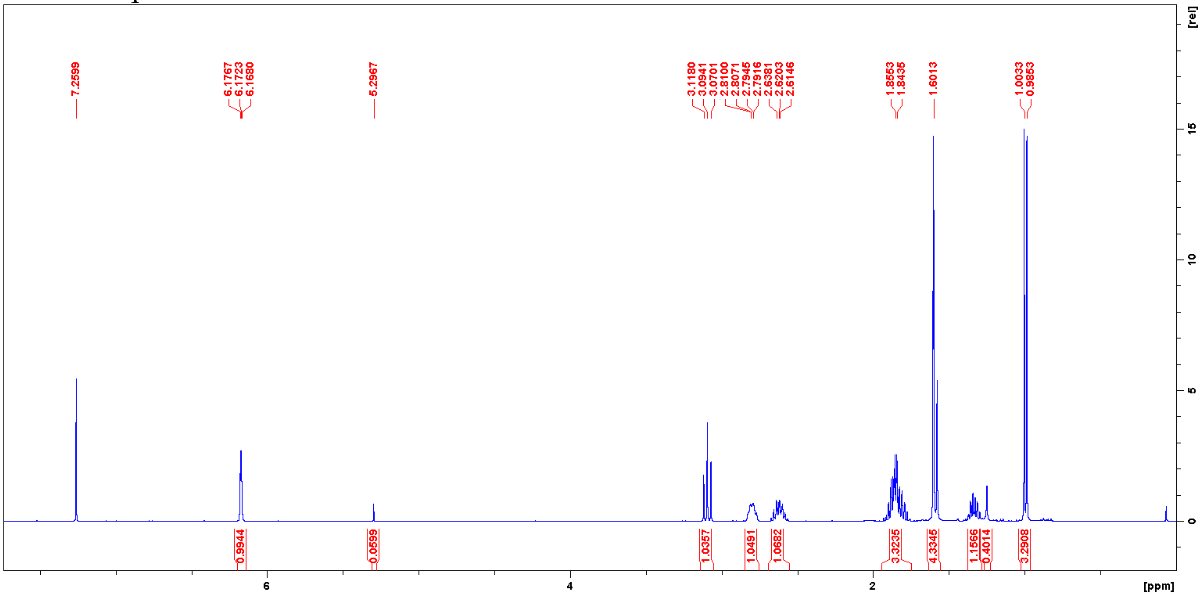


*Cis*-*cis*-nepetalactol $^1$H-NMR in $CDCl_3$

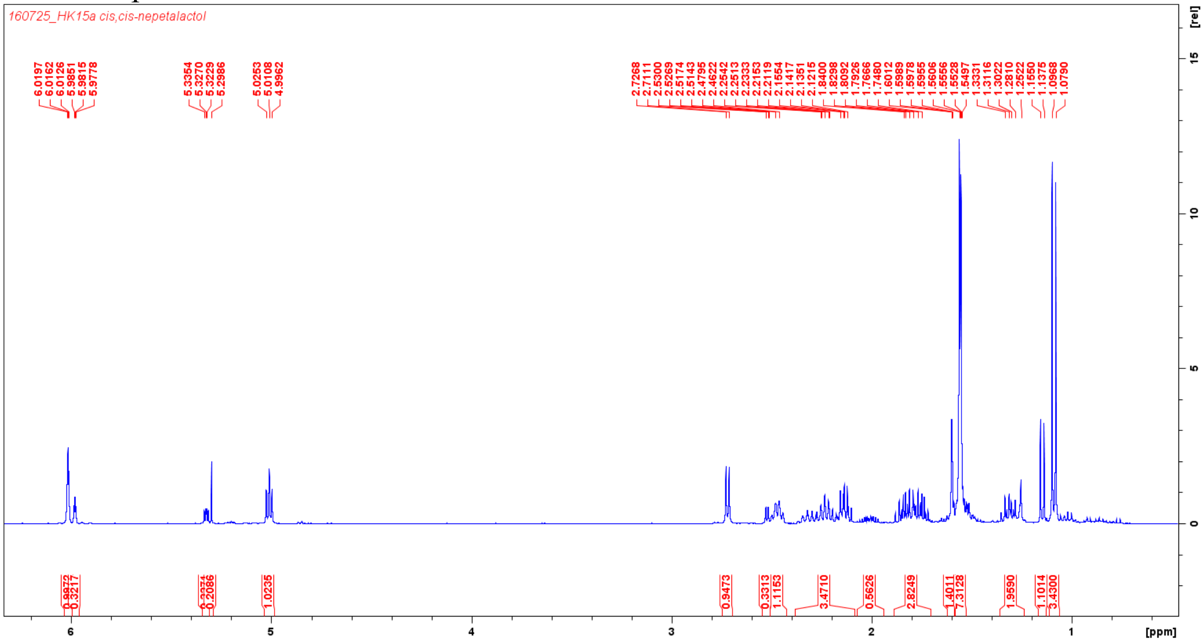

*Trans-trans*-nepetalactone $^{1}$H-NMR in $CDCl_3$

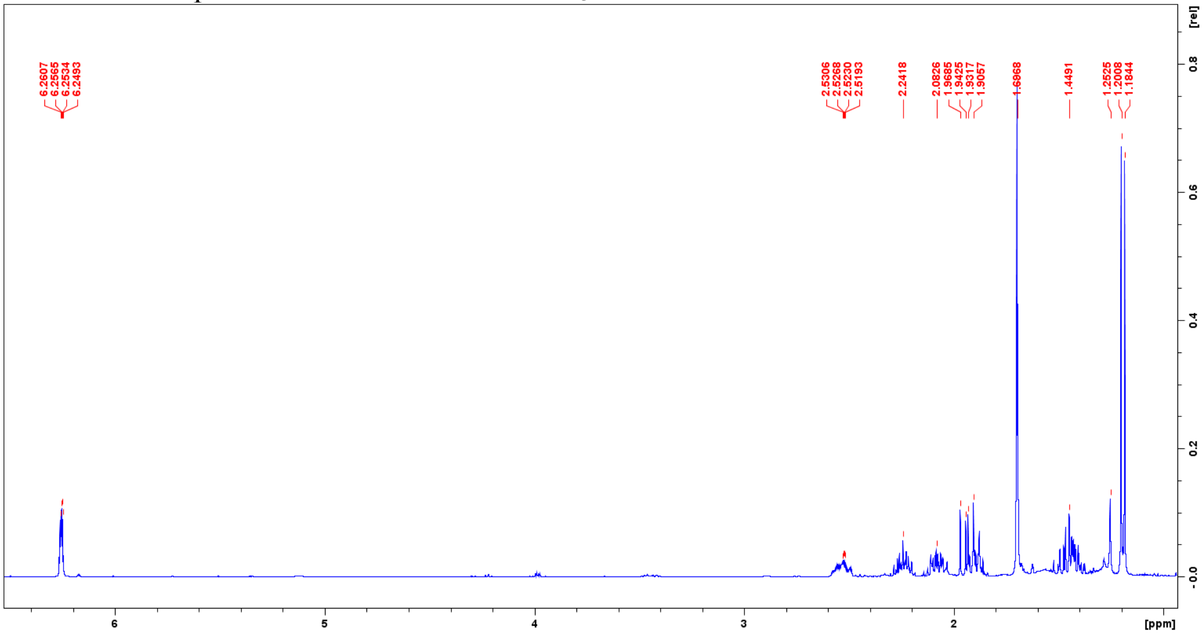


*Trans-trans*-iridodial $^{1}$H-NMR in $CDCl_3$

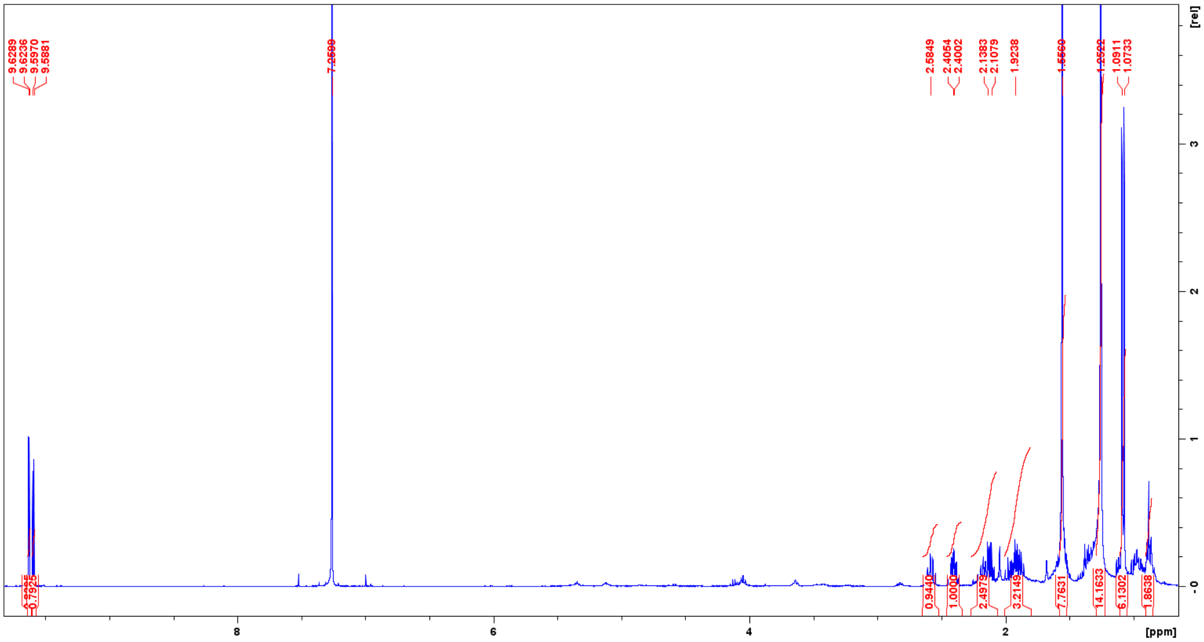

*Trans-trans*-iridodial $^{13}$C-NMR in $CDCl_3$

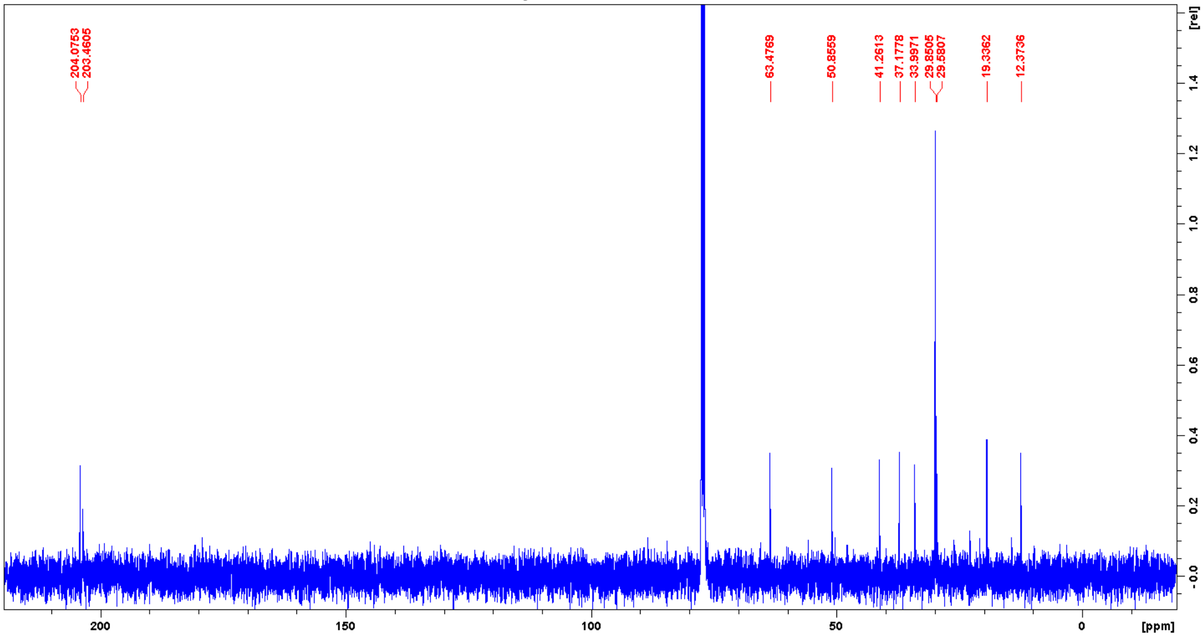

*Trans-trans*-iridodial $^1$H-COSY-NMR in $CDCl_3$

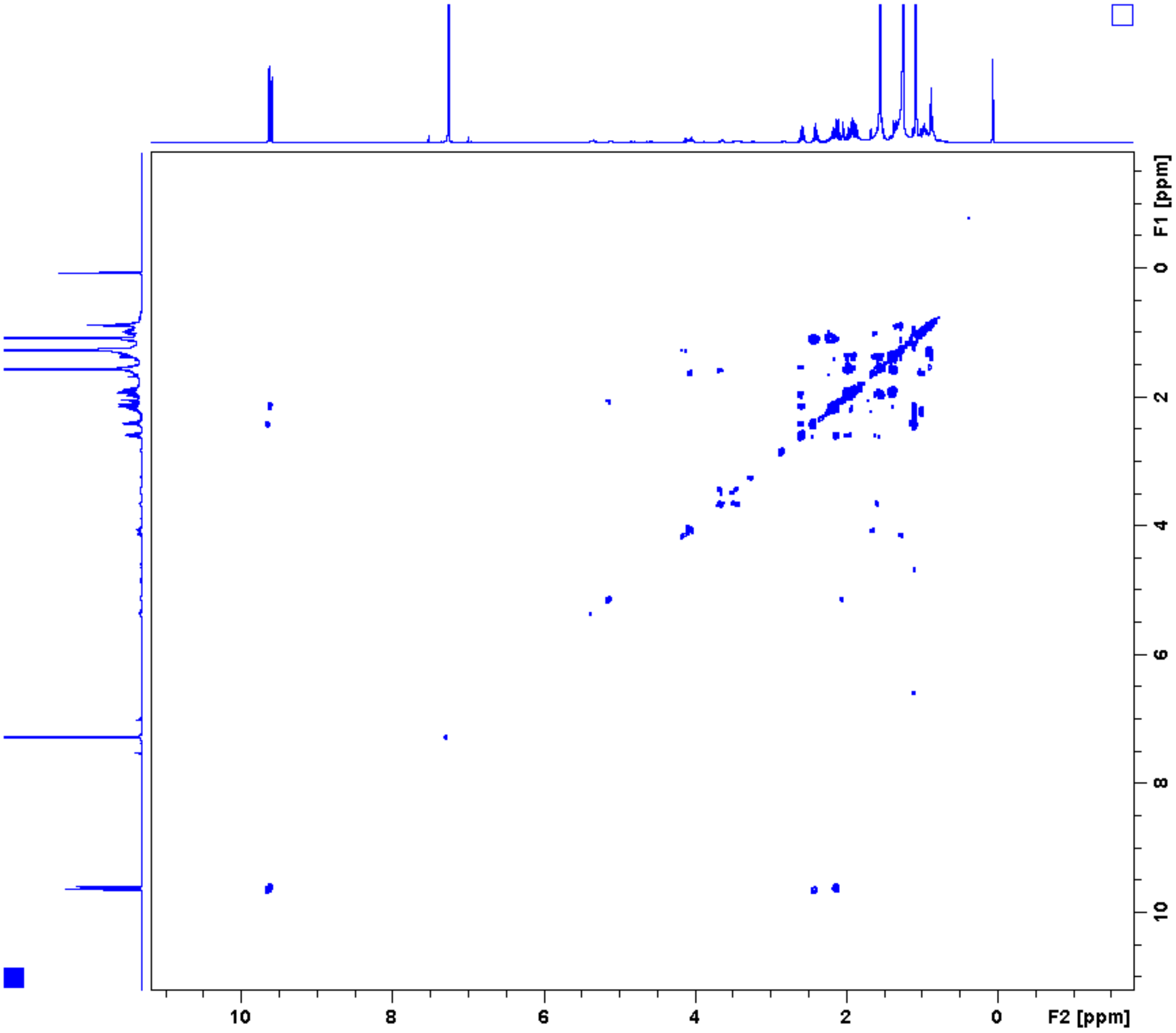

*Trans-trans*-iridodial $^{1}H/^{13}C$-HSQC-NMR in $CDCl_3$

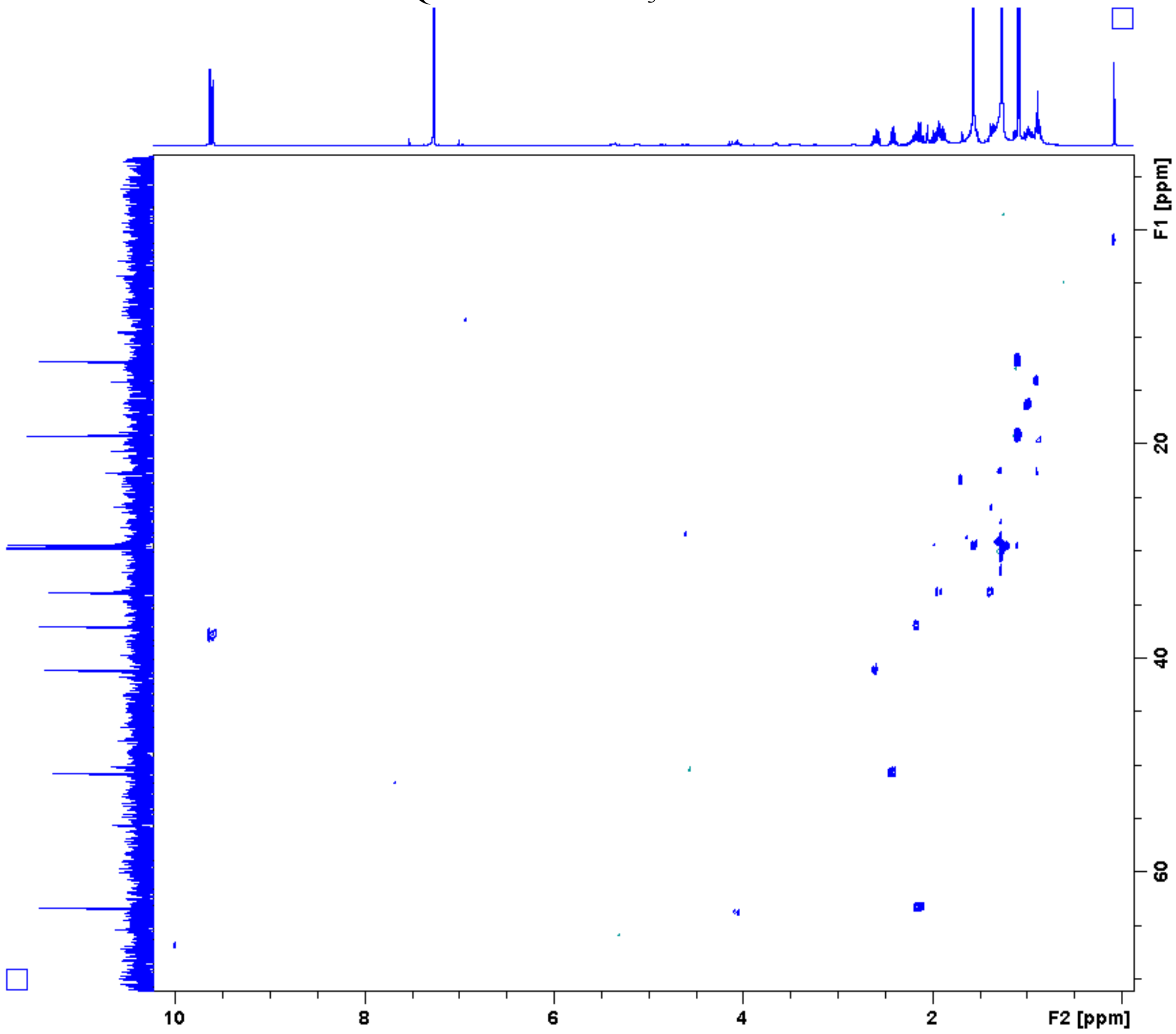

Supplement: Supplemental Data [file 10.1074_M117.800979_jbc.M117.800979-1.pdf]
